# Atomic force microscopy data of novel high-*k* hydrocarbon films synthesized on Si wafers for gate dielectric applications

**DOI:** 10.1016/j.dib.2020.105652

**Published:** 2020-04-30

**Authors:** Jihwan Kwon, Dong-Ok Kim, Sangyeob Lee, Eui-Tae Kim

**Affiliations:** a Department of Materials Science & Engineering, Chungnam National University, Daejeon, 34134, Republic of Korea; b Department of Materials Science and Engineering, Hanbat National University, Daejeon, 34158, Republic of Korea

**Keywords:** Hydrocarbon, High-*k* dielectrics, Chemical vapor deposition, Atomic force microscopy

## Abstract

This article presents data obtained from the atomic force microscopy (AFM) images of ultrathin high-*k* hydrocarbon (HC) films. The high-*k* HC films were synthesized on Si(100) wafers at various growth temperatures by using inductively-coupled plasma chemical vapor deposition with CH_4_ gas and a gas mixture consisting of 10% H_2_ and 90% Ar. The AFM images were obtained by tapping mode. The AFM results provide the surface topography, roughness, and thickness of the HC films as a function of growth temperature, which are essential data for high-*k* gate dielectrics of metal-insulator-semiconductor device applications. This data article is related to the article entitled, “Novel high-*k* gate dielectric properties of ultrathin hydrocarbon films for next-generation metal-insulator-semiconductor devices” (Kim et al., 2020) [1].

Specifications Table

**Table:** 

Subject	Material science
Specific subject area	Dielectric surface, atomic force microscopy (AFM)
Type of data	Image and graph (AFM)
How data were acquired	AFM (Asylum Research, MFP-3D)
Data format	Raw and analyzed
Parameters for data collection	The surface topography and thickness of high-k hydrocarbon (HC) films synthesized on Si(100) at various growth temperatures of inductively-coupled plasma chemical vapor deposition (ICP CVD)
Description of data collection	Tapping mode of AFM in ambient environment
Data source location	Chungnam National University, Daejeon 34134, Republic of Korea
Data accessibility	The data are with this article
Related research article	Author's name: Dong-Ok Kim et al. Title: Novel high-*k* gate dielectric properties of ultrathin hydrocarbon films for next-generation metal-insulator-semiconductor devices Journal: Carbon DOI:10.1016/j.carbon.2019.11.019

## Value of the data

• The thickness of the high-*k* gate dielectric films should be controlled on a scale of several nm for sub-10 nm node semiconductor technology. Therefore, it is also critical to control the morphology and pinholes of the high-*k* dielectric films in order to maintain a high-capacitance density with a low leakage current. These AFM results provide important information indicating that novel high-*k* HC films can be applied to next-generation metal-insulator-semiconductor (MIS) devices.
• Detailed surface information of the high-*k* HC films can be useful to the researchers who study and seek new high-*k* gate dielectrics for next-generation sun-10 nm node Si and organic semiconductor technologies.
• The AFM data, as a function of growth temperature, provide a new process along with the processing conditions of the high-*k* HC films for various applications, such as MIS devices and supercapacitors.

## 1 Data Description

**Fig. 1:**
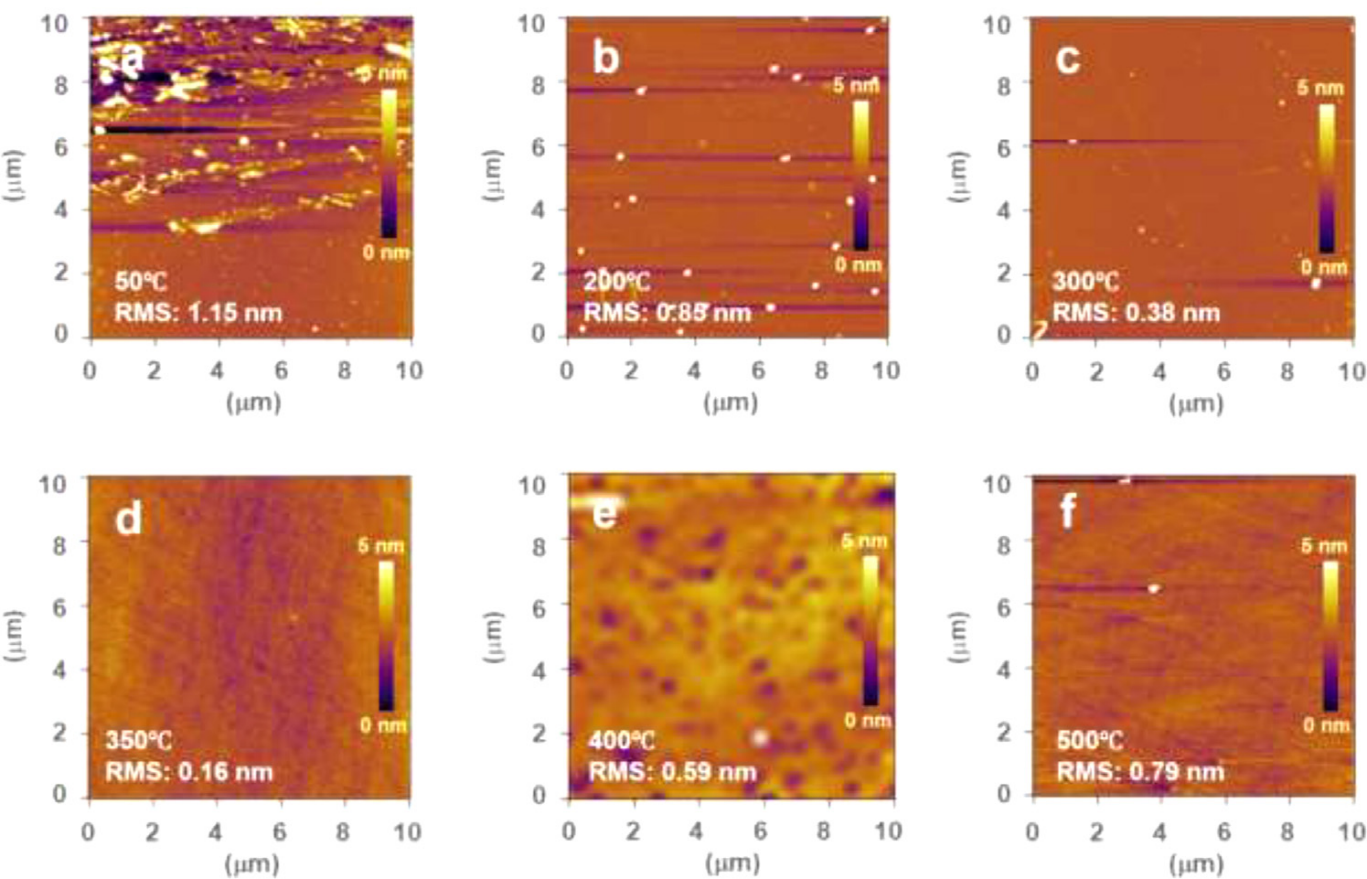
10 μm × 10 μm AFM images of the HC films synthesized on Si(100) wafers at various growth temperatures: (a) 50°C, (b) 200°C, (c) 300°C, (d) 350°C, (e) 400°C, and (f) 500°C.

**Fig. 2:**
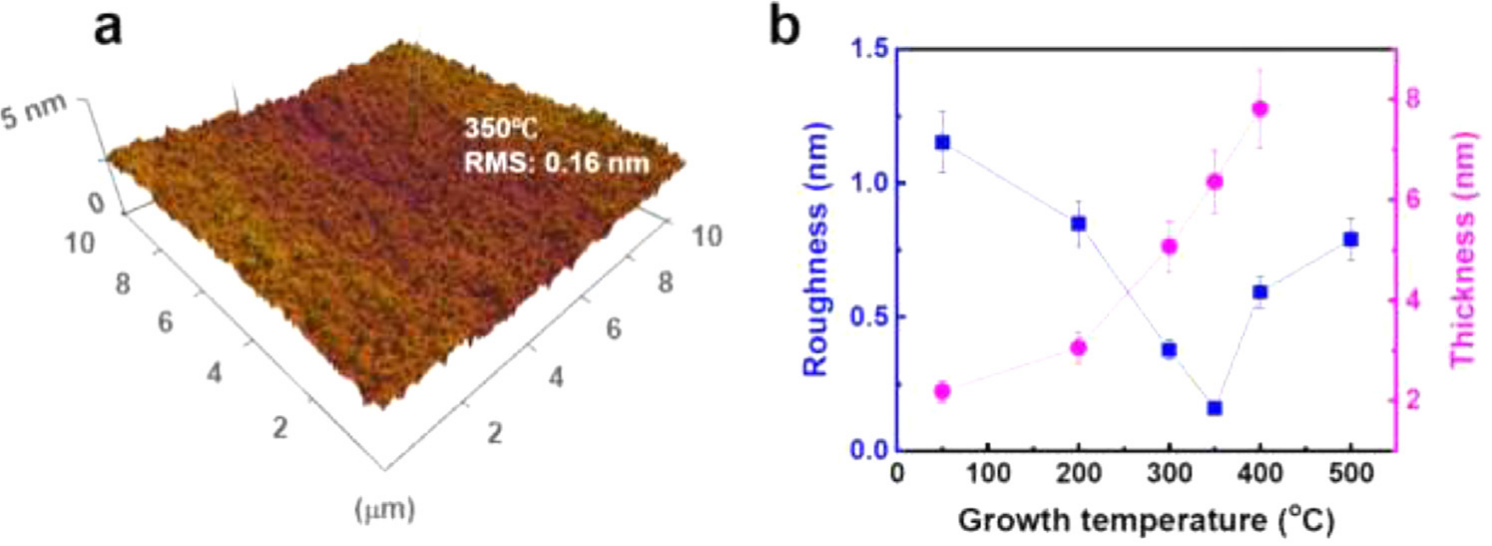
(a) 3D surface topography of the HC film synthesized at 350°C. (b) Thickness and RMS roughness values of HC films as a function of growth temperature (corresponding to AFM images of Figure 1).

This data article provides AFM surface images of high-*k* HC films synthesized by ICP CVD technique. Figure 1 shows the 10 μm × 10 μm AFM images of HC films grown on Si(100) wafers at various growth temperatures (50°C, 200°C, 300°C, 350°C, 400°C, and 500°C). As shown in Figure 1(a), the HC film synthesized at 50°C exhibited the roughest surface morphology. As the growth temperature increased, the surface became gradually smoother. The HC film synthesized at 350°C showed the smoothest surface (Figure 1(d)). With further increase in the growth temperature, the roughness of the HC films increased. Figure 2(a) shows a three-dimensional surface topography of the HC film synthesized at 350°C, which exhibits a uniform, pinhole-free, and smooth surface. In fact, the highest *k* value of the HC films was achieved at 350°C [1]. Figure 2(b) shows the thickness and root-mean-square (RMS) roughness values of the HC films as a function of growth temperature. The thickness of HC films increased almost linearly from 2.2 nm at 50°C to 7.8 nm at 400°C. The HC film synthesized at 50°C yielded the highest RMS roughness value of 1.15 nm. The RMS roughness value continuously decreased with increasing growth temperature to the minimum value of 0.16 nm for 350°C and then increased to 0.79 nm for 500°C.

## 2 Experimental Design, Materials, and Methods

The synthesis of HC films was carried out in a hot-wall tubular ICP CVD chamber. The ICP CVD system has been described in further detail elsewhere [2]. The HC films were synthesized on Si(100) wafers for 30 min. The Si wafers were pre-cleaned by a solvent clean, which involved dipping in acetone for 10 min, followed by dipping in methanol for 5 min, and rinsing in de-ionized (DI) water. Then, the Si wafers were dipped in a 10% HF solution for 5 min, followed by washing with DI water in order to remove the native oxide. The pre-treated Si wafers were loaded onto the CVD chamber. The chamber temperature was increased to a desired growth temperature with a continuous flow of a gas mixture consisting of 10% H_2_ and 90% Ar. The HC films were synthesized with CH_4_ gas and a gas mixture of 10% H_2_ and 90% Ar at rates of 1 and 100 standard cubic centimeters per minute, respectively, at various growth temperatures (50°C, 200°C, 300°C, 350°C, 400°C, and 500°C). The ICP power and growth pressure were fixed at 600 W and 1 Torr, respectively. Atomic-force microscopy (AFM; Asylum Research, MFP-3D) was conducted to investigate the surface morphology of each HC films.

## Appendix Supplementary materials

Image, application 1

## References

[1] Kim D.O., Hong H.K., Seo D.B., Trung T.N., Hwang C.C., Lee Z., Kim E.T. (2020). Novel high-*k* gate dielectric properties of ultrathin hydrocarbon films for next-generation metal-insulator-semiconductor devices. Carbon.

[2] Nang L.V., Kim E.T. (2012). Controllable synthesis of high-quality graphene using inductively-coupled plasma chemical vapor deposition. J. Electrochem. Soc..

